# Supplementary material for: Acute Bronchitis and Bronchiolitis Infection in Children with Asthma and Allergic Rhinitis: A Retrospective Cohort Study Based on 5,027,486 Children in Taiwan
Source: Viruses. 2023 Mar 22;15(3):810. doi: 10.3390/v15030810 (PMC10054660; doi:10.3390/v15030810)
Supplement: Supplementary file 1 [file viruses-15-00810-s001.zip › viruses-2279072-supplementary.pdf]

Supplement Table S1. Incidence number and rate of acute bronchitis, asthma cohort to non-asthma cohort hazard ratio and allergic rhinitis cohort to non-allergic rhinitis cohort hazard ratio by urbanization level of resident and parental job

	Asthma					
	Yes (N=192126)		No (N=192126)		Hazard ratio (95% CI)	
Variable	n	Rate	n	Rate	Crude	Adjusted
All	166527	525.1	143977	236.0	1.78 (1.77-1.80)	1.82 (1.80-1.83)
Urbanization						
1, highest	38051	448.8	34168	213.5	1.74 (1.71-1.77)	1.76 (1.77-1.82)
2	55765	540.1	45254	240.5	1.79 (1.77-1.81)	1.83 (1.80-1.85)
3	55177	558.8	50810	248.9	1.79 (1.76-1.81)	1.81 (1.79-1.84)
4	17534	578.0	13745	237.2	1.86 (1.82-1.90)	1.86 (1.82-1.90)
Parental job						
Public Employees	12995	452.5	11371	232.8	1.66 (1.62-1.71)	1.72 (1.68-1.77)
Business	87566	563.3	73728	260.6	1.74 (1.73-1.76)	1.79 (1.77-1.80)
Industry	24480	514.6	20844	238.6	1.76 (1.72-1.79)	1.81 (1.78-1.85)
Agriculture	13960	469.5	11839	217.5	1.73 (1.68-1.77)	1.73 (1.69-1.78)
Fishery	5629	559.0	4951	249.2	1.79 (1.72-1.86)	1.82 (1.75-1.89)
Other	21897	480.6	21244	181.8	2.04 (2.00-2.08)	2.07 (2.03-2.11)
	Allergic rhinitis					
	Yes (N=1062903)		No (N=1062903)		Hazard ratio (95% CI)	
	n	Rate	n	Rate	Crude	Adjusted
All	898468	322.4	768339	169.9	1.61 (1.60-1.61)	1.68 (1.68-1.69)
Urbanization						
1, highest	246689	288.2	180784	154.9	1.60 (1.59-1.61)	1.65 (1.64-1.66)
2	287237	329.1	239453	172.2	1.62 (1.61-1.63)	1.69 (1.68-1.69)
3	292292	344.3	270437	177.9	1.62 (1.61-1.63)	1.70 (1.69-1.70)
4	72250	345.9	77665	175.1	1.63 (1.62-1.65)	1.71 (1.70-1.73)
Parental job						
Public Employees	90154	282.3	62200	158.9	1.55 (1.53-1.56)	1.62 (1.61-1.64)
Business	469670	338.1	371634	186.8	1.55 (1.54-1.56)	1.63 (1.62-1.63)
Industry	137646	317.1	116754	169.8	1.59 (1.57-1.60)	1.68 (1.66-1.69)
Agriculture	68166	295.4	72793	156.4	1.59 (1.58-1.61)	1.69 (1.67-1.70)
Fishery	29174	348.5	27483	183.7	1.59 (1.56-1.61)	1.66 (1.63-1.68)
Other	103658	314.5	117475	140.3	1.86 (1.84-1.87)	1.99 (1.97-2.01)

Rate, per 1000 person-years; CI, confidence interval; other, joblessness or financial fragility.

Supplement Table S2. Incidence of acute bronchiolitis, asthma cohort to non-asthma cohort hazard ratio and allergic rhinitis cohort to non-allergic rhinitis cohort hazard ratio by urbanization level of resident and parental job

	Asthma					
	Yes (N=192126)		No (N=192126)		Hazard ratio (95% CI)	
Variable	n	Rate	n	Rate	Crude	Adjusted
All	54222	42.7	41365	28.5	1.45 (1.43-1.47)	1.50 (1.48-1.52)
Urbanization						
1, highest	11498	38.8	9235	25.8	1.44 (1.40-1.48)	1.56 (1.52-1.61)
2	18008	43.9	13068	29.3	1.44 (1.40-1.47)	1.50 (1.46-1.53)
3	18973	45.3	15522	31.1	1.42 (1.39-1.45)	1.46 (1.43-1.49)
4	5743	39.8	3540	23.8	1.62 (1.56-1.69)	1.58 (1.52-1.65)
Parental job						
Public Employees	3839	34.4	3106	25.8	1.33 (1.26-1.39)	1.43 (1.36-1.50)
Business	29686	48.2	22436	32.9	1.41 (1.39-1.44)	1.49 (1.46-1.52)
Industry	7529	40.1	5902	28.4	1.38 (1.33-1.42)	1.46 (1.42-1.52)
Agriculture	4245	31.1	2917	20.2	1.51 (1.44-1.59)	1.55 (1.48-1.62)
Fishery	1852	43.6	1438	28.4	1.45 (1.36-1.56)	1.44 (1.34-1.54)
Other	7071	40.4	5566	22.5	1.70 (1.64-1.76)	1.73 (1.67-1.79)

	Allergic rhinitis					
	Yes (N=1062903)		No (N=1062903)		Hazard ratio (95% CI)	
Variable	n	Rate	n	Rate	Crude	Adjusted
All	253865	29.5	191399	20.1	1.42 (1.41-1.43)	1.46 (1.45-1.47)
Urbanization						
1, highest	66196	26.8	42921	18.5	1.42 (1.40-1.44)	1.43 (1.41-1.45)
2	80280	29.6	59582	20.3	1.41 (1.39-1.42)	1.45 (1.43-1.46)
3	87990	32.4	71804	22.0	1.42 (1.40-1.43)	1.47 (1.46-1.49)
4	19399	27.4	17092	17.1	1.53 (1.50-1.56)	1.57 (1.54-1.60)
Parental job						
Public Employees	22958	23.5	14328	17.3	1.35 (1.32-1.37)	1.41 (1.38-1.44)
Business	139856	32.8	100568	23.7	1.35 (1.34-1.36)	1.40 (1.39-1.41)
Industry	37374	28.2	27692	19.0	1.43 (1.40-1.45)	1.50 (1.47-1.52)
Agriculture	17470	22.9	15464	14.8	1.50 (1.46-1.53)	1.56 (1.53-1.59)
Fishery	8430	30.9	6334	18.8	1.56 (1.51-1.62)	1.63 (1.57-1.68)
Other	27777	27.5	27013	16.9	1.57 (1.53-1.59)	1.66 (1.63-1.69)

Rate, per 1000 person-years; CI, confidence interval; other, joblessness or financial fragility.

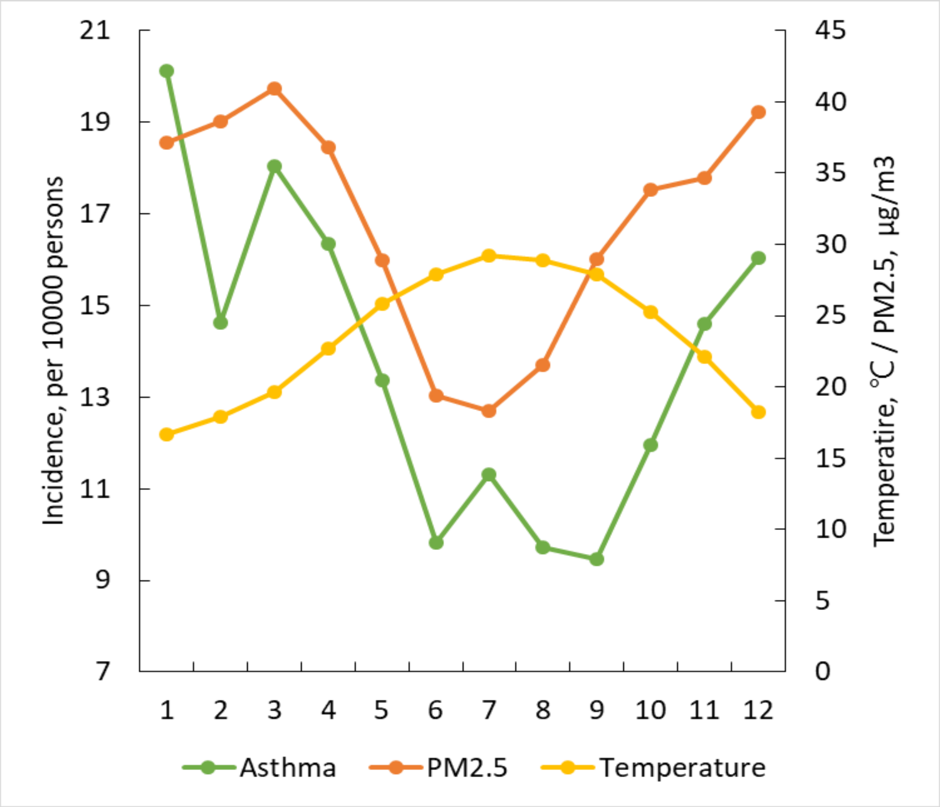

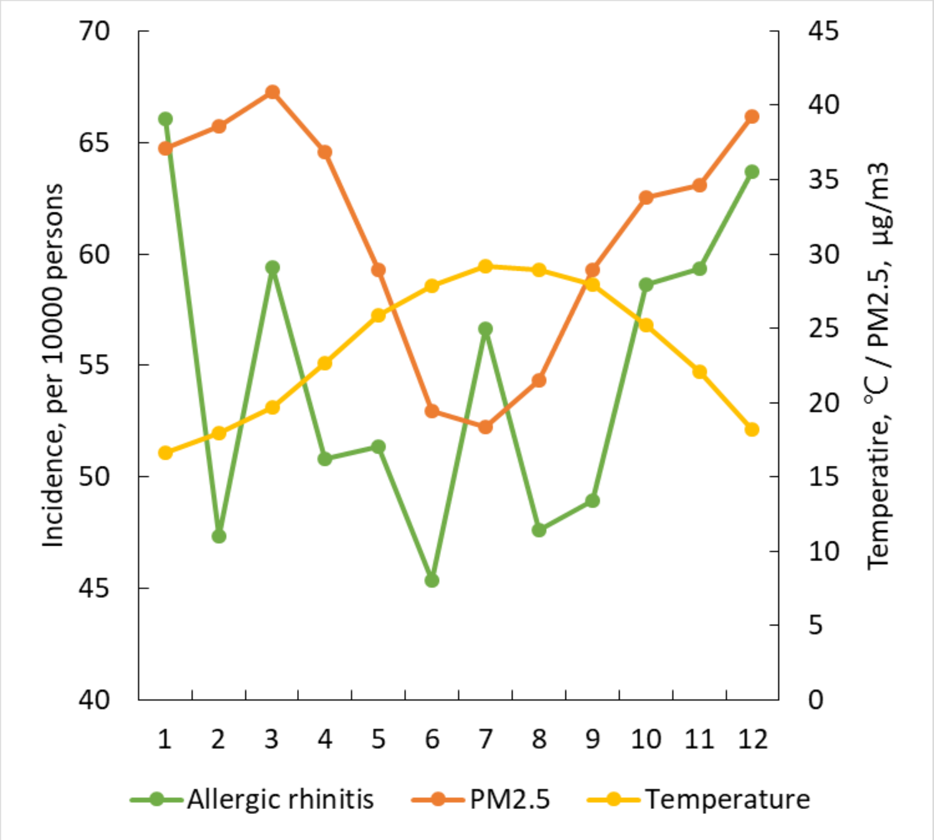

Supplement Figure S1. Monthly average incident childhood asthma and allergic rhinitis by monthly average temperature and $PM_{2.5}$ level in 2006–2016 in Taiwan
